# Behavioral and Neural Mechanisms Underlying Sensory–Motor Integration

**DOI:** 10.3390/brainsci14080812

**Published:** 2024-08-14

**Authors:** Valentina Bianco, Renato Borgatti, Marika Berchicci

**Affiliations:** 1 Department of Brain and Behavioral Sciences, University of Pavia, 27100 Pavia, Italy; renato.borgatti@mondino.it; 2 Child Neurology and Psychiatry Unit, IRCCS Mondino Foundation, 27100 Pavia, Italy; 3 Department of Psychology, University “G. d’Annunzio”, 66100 Chieti Scalo, Italy; m.berchicci@gmail.com

Sensory–motor integration represents a complex process requiring proper orchestration among multiple sources of sensory information to ensure the best task-related motor output. This process relies on the proper integration of visual, auditory, and haptic perceptual inputs from primary and associative sensory areas and pre-motor and motor cortical areas. The present SI received more than 10 valuable contributions, which nicely highlighted the nuances of the advances related to this intricate and multifaceted topic, ranging from basic to clinical research.

Berchicci and co-authors addressed the electrophysiological correlates of proactive control processing preceding the execution of different visual discriminative tasks, with particular focus on the modulation of specific anticipatory event-related potentials (ERPs). Namely, they explored the sensory, cognitive, and motor processing occurring during the preparation of tasks by tapping into response selection (i.e., the ability to react to a certain stimulus) vs. response inhibition (i.e., the ability to refrain from reacting to a specific stimulus). Notably, the findings showed that the amplitude of the ERPs associated with proactive motor control was maximal during the execution of the task prompting response inhibition.

Su et al. leveraged functional near-infrared spectroscopy (fNIRS) to test the existence of different cortical activations during meaningless actions, tool use, and pantomime actions in children with or without autism spectrum disorder (ASD). This latter group showed impaired praxis performance and hyperactivation of the right inferior parietal lobe (IPL) during all types of gestures, consistent with the atypically reduced left-hemispheric dominance observed in ASD. Critically, they provide evidence of atypical proprioceptive and visuospatial processing in ASD and propose interesting fNIRS-related neurobiomarkers.

The behavioral study in school-aged children conducted by Alhamdan and colleagues investigated the relation between the performance of visual, auditory, and multisensory motor tasks and individual measures of receptive language (i.e., understanding the meaning of words) and expressive vocabulary (i.e., outsourcing the meaning of words) development. Leveraging Bayesian and multi-regression analyses, they showed that the complexity of expressive vocabulary abilities, which require the combination of sensory processing with verbal outputs, is more related to efficient reactivity to multisensory stimuli than receptive vocabulary skills.

The ERP study by Di Russo and Bianco tested the existence of different EEG markers associated with interference and facilitation during a typical Stroop test. Interference occurs in identifying the print color of a word when the word represents the name of another color. Facilitation occurs when the word is the same as the print color. Interestingly, the authors used a novel experimental design in which neutral trials were separately intermixed with either congruent or incongruent trials. Overall, ERP findings suggest that the facilitation induced by the congruency and the interference induced by the incongruency reflect the result of independent mechanisms rather than decreased or increased activity of neural areas (e.g., anterior cingulate cortex, ACC) involved in response conflict detection.

Given the relation between sensory-motor integration and postural control, the clinical study by Leodori and co-authors explored the effects of the interaction between the typical pharmacological dopaminergic treatment and the innovative deep brain stimulation (DBS) of the subthalamic nucleus (STN) in advanced Parkinson’s disease (PD) patients. The findings showed that postural instability and risk of falls, assessed using a stabilometric platform, were promisingly attenuated by DBS-STN when combined with dopaminergic medication. Similarly, the study of the interaction between pharmacological treatment and DBS-STN was one of the main objectives of Heß et al., together with the evaluation of how impaired plantar cutaneous vibration perception can contribute to axial motor symptoms in PD patients. Crucially, they showed that DBS-STN, combined with anti-parkinsonian medication, improves the plantar cutaneous vibration perception of patients compared to medication alone. Overall, these two studies provide promising evidence for the integration of DBS in therapeutic strategies to improve sensory and motor performance in PD patients.

Fiori et al. addressed the effects of viewing faces expressing basic emotions or neutral emotions on cortico-spinal excitability (CSE). Indeed, the motor system is promptly activated by the perception of emotional faces to prepare for the proper strategy, as in the case of the fight or flight reaction. Leveraging single-pulse transcranial magnetic stimulation (sp-TMS), motor-evoked potentials (MEPs) were elicited from the left motor cortex. The authors found that observing happy, disgusted, angry, and fearful faces induced a significant increase in CSE. Overall, this provides interesting evidence that only observing emotional faces with rewarding or threat values impacts CSE.

In the behavioral study by Bianco and co-authors, the aim was to assess the temporal deployment of contextual modulation on action prediction with the increasing availability of kinematic cues during action observation. Supporting longstanding predictive coding views of action understanding, they showed the existence of a continuous “advantage” of contextual priors when guessing action intentions, even in the case of fully available kinematic information. Contextual (i.e., prior experience) and kinematic (i.e., sensorial evidence) information are thus continuously integrated during action observation at all stages of action deployment. Further, in line with previous evidence showing an association between autism and social deficits, they also provide evidence of a dysfunctional integration between context and kinematics cues during action prediction in individuals with high levels of autistic traits.

In a clinical pilot study, Schranz and Seo investigated to what extent deficits in cortical sensory-motor integration could contribute to impaired grip force direction control in patients affected by stroke. Sp-TMS was applied to the affected hemisphere, preceded by median nerve stimulation, allowing the measure of short-latency afferent inhibition (SAI). The authors showed that patients with higher SAI (i.e., higher afferent inhibition) had a lower deviation of the grip force direction from the normal direction (i.e., better control). Despite the limited sample size of the study, the results suggest that paired associative stimulation should be encouraged in neurorehabilitation contexts for movement disorders.

The developmental study conducted by Vitali and co-authors leveraged EEG to address the relation between cortical oscillations and the non-nutritive sucking behavior (NNS), an endogenous rhythmic movement of infants with origin in the sucking central pattern generator in the brainstem. Focusing on beta synchronization, authors analyzed specific time bins ranging from the pre-movement to the post-movement epoch and acknowledged two phases involved in sucking regulation: an early one, occurring a few seconds before the NNS, in which the activity positively correlated with sucking frequency, and a late one just before the NNS, in which the activity negatively correlated with sucking frequency. This specific temporal dynamic of beta modulation suggests that the complexity of the coordination among sensory-motor networks is already present in newborns.

The topic of the paper by Lazzari and co-authors relates to deficits in sensory-motor integration observed in individuals who stutter. Namely, they used different non-verbal sensory-motor synchronization tasks to explore tapping performance following delayed auditory feedback with the instruction to align with or ignore it. The findings showed that, compared to controls, individuals who stutter tend to refrain from adapting their performance to the feedback. Interestingly, this study provides further evidence that stuttering goes beyond deficits in linguistic networks but involves more complex circuits related to proper perception-action coupling.

The last contribution to this Special Issue, i.e., Grumi et al., explored to what extent different types of parental verbal and touch stimulations can influence attention orientation, considered a product of early sensory-motor integration in infants with visual impairment. Participants performed a face-to-face still-face (FFSF) task comprising three episodes: a first phase of face-to-face play interaction (play), a second phase of still-face (still), and again a phase of face-to-face play interaction (reunion). The authors showed that maternal verbal and tactile stimuli modulate gaze orientation differently in visually impaired infants and controls, with the latter being more prone to direct attention to the mother during the play phase of the task. This study highlights the importance of sensory-motor integration for social development and provides support for family-centered interventions in clinical contexts.

The present Special Issue highlights the multifaceted nature of sensory-motor integration in a broad spectrum of clinical and non-clinical contexts, testifying to the complexity of the phenomenon. A better understanding of the neural networks involved in the integration between multimodal stimuli and motor behavior in developmental children and in adults’ populations might aid future rehabilitative interventions [1] aimed at improving not only sensory perception and motor control [2] but also social skills [3], heavily related to intact sensory-motor integration.
